# Temperature and Irrigation-Frequency Treatments Modulate Phytochemical Profiles and Antioxidant Capacity in *Guzmania stenostachya* and *Werauhia macrochlamys*

**DOI:** 10.3390/molecules31193395

**Published:** 2026-09-24

**Authors:** Viviana Carrasco, Deilyn Quiel, Yovelis Sandoval-Moreno, Darielis Lezcano, Henry Velásquez, Edilia de la Rosa Manzano, Diana C. Gómez González

**Affiliations:** 1 Instituto Interdisciplinario de Investigación e Innovación, Vicerrectoría de Investigación y Posgrado, Universidad Autónoma de Chiriquí, David 0427, Chiriquí, Panama; viviana.carrasco@unachi.ac.pa; 2 Centro de Investigación en Bioquímica y Química Aplicada, Facultad de Ciencias Naturales y Exactas, Universidad Autónoma de Chiriquí, David 0427, Chiriquí, Panama; deilyn.quiel@unachi.ac.pa; 3 Ciencias Químicas con Énfasis en Inocuidad Alimentaria, Facultad de Ciencias Naturales y Exactas, Universidad Autónoma de Chiriquí, David 0427, Chiriquí, Panama; 4 Departamento de Química Analítica, Universidad de Panamá, Panama City 3366, Panamá Province, Panama; yovelis-v.sandoval-m@up.ac.pa; 5 Escuela de Biología, Facultad de Ciencias Naturales y Exactas, Universidad Autónoma de Chiriquí, David 0427, Chiriquí, Panama; darielis.lezcano@unachi.ac.pa (D.L.); henry.velasquez@unachi.ac.pa (H.V.); 6 Instituto de Ecología Aplicada, Universidad Autónoma de Tamaulipas, Centro de Educación Continua FIC, Ciudad Victoria 87149, Tamaulipas, Mexico; ermanzano@docentes.uat.edu.mx; 7 Facultad de Ingeniería y Ciencias, Universidad Autónoma de Tamaulipas, Centro Universitario Adolfo López Mateos, Ciudad Victoria 87000, Tamaulipas, Mexico

**Keywords:** Bromeliaceae, abiotic stress, GC-MS, DPPH, ABTS, climate change, phenolic compounds, phytochemistry

## Abstract

Information on how temperature and irrigation-frequency treatments modify secondary metabolism in Bromeliaceae remains limited, particularly for poorly studied species such as *Guzmania stenostachya* and *Werauhia macrochlamys.* This study integrated the characterization of the phytochemical profile, total phenolic content (TPC), and antioxidant capacity to evaluate the responses of both species under controlled temperature and irrigation-frequency conditions. Hydroethanolic extracts were analyzed using the Folin–Ciocalteu, ABTS, and DPPH assays and gas chromatography–mass spectrometry (GC-MS). Species, treatment, and their interaction significantly influenced most of the responses evaluated. Under temperature treatments, *W. macrochlamys* maintained higher TPC and antioxidant capacity values, although both decreased with increasing temperature, whereas *G. stenostachya* exhibited a nonlinear response and changes in its chemical profile. Irrigation-frequency treatments resulted in contrasting patterns between species and exposure times. Overall, the results showed that the phytochemical profile depended on the species and on temperature and irrigation-frequency treatments, expanding current knowledge of the responses of these Bromeliaceae to abiotic stress. The observed chemical diversity also suggests biotechnological and nutraceutical potential that may vary with environmental factors associated with climate change.

## 1. Introduction

Climate change refers to alterations in the environmental conditions of the planet resulting from both natural processes and human activities and it is manifested globally through warming of the Earth’s surface, changes in climate patterns, and the intensification of extreme events [1]. According to the Intergovernmental Panel on Climate Change [2], climate models project that the global mean temperature will reach 1.5 °C before 2040, even under low-emission scenarios, and that increases exceeding 4 °C could be recorded by the end of the century if high emissions persist, representing a significant challenge for terrestrial ecosystems.

In this context, increasing temperatures and decreasing water availability constitute abiotic stress factors that directly affect plant growth and development by altering essential physiological and metabolic processes. Thermal stress and drought induce the generation of reactive oxygen species (ROS), causing cellular damage, impairment of the photosynthetic machinery, decreased antioxidant capacity, and reduced physiological performance, while also affecting the uptake and transport of essential nutrients [3].

Plant adaptive responses to these stress conditions include the activation of both enzymatic and non-enzymatic antioxidant systems, as well as the accumulation of secondary metabolites. In particular, phenolic compounds play a central role in tolerance to abiotic stress because of their ability to limit oxidative damage. Therefore, the regulation of these metabolites, including flavonoids, anthocyanins, and lignins, is crucial for limiting oxidative damage induced by factors such as thermal and water stress [4].

Within this framework, the Bromeliaceae family represents a plant group of particular interest because of its broad geographical distribution, extending from the southern United States to South America, with one species native to West Africa, as well as its ability to adapt to a wide variety of environments, ranging from arid regions to tropical rainforests and across elevational ranges reaching approximately 4000 m a.s.l. [5]. This family exhibits high diversity in the Neotropical region, comprising approximately 3800 species distributed among 82 genera, many of which are epiphytic [6].

The Bromeliaceae family has considerable ecological and cultural value, performing key functions as microhabitats for diverse organisms [7,8] and being used for ornamental, food, ritual, and medicinal purposes. Regarding the latter, bromeliads have been associated with antioxidant, antibacterial, and anti-inflammatory properties, suggesting considerable potential as sources of secondary metabolites that remains poorly explored [9,10]. Because bromeliads are sensitive to fluctuations in temperature and water availability in their natural habitats [5,9], changes in these environmental factors may influence their physiological and metabolic responses. Nevertheless, available information on how temperature and irrigation-frequency treatments affect total phenolic content, phytochemical profiles, and antioxidant capacity in Bromeliaceae remains limited.

*Guzmania stenostachya* L.B.Sm. and *Werauhia macrochlamys* (Mez & Wercklé) J.F. Morales are epiphyte plants distributed in South America, and Central America including Costa Rica, Panamá [11], and represent different life forms within Bromeliaceae. *W. macrochlamys* belongs to the group of species commonly referred to as “tank” bromeliads, which store water in a rosette formed by the overlapping bases of their broad leaves, whereas *G. stenostachya* belongs to the group of “atmospheric” bromeliads, which generally have smaller structures, abundant trichomes, and adaptations for water conservation [12,13].

In this regard, to the best of our knowledge, no studies have been published integrating the characterization of the phytochemical profiles of *G. stenostachya* and *W. macrochlamys* with the determination of total phenolic content and the evaluation of antioxidant capacity. Likewise, although morphological and germination responses of some species to increasing temperature have been documented [13,14], it remains unknown how their phytochemical composition and antioxidant capacity vary under abiotic stress conditions, particularly temperature and irrigation-frequency treatments.

Therefore, the present study aimed to characterize and compare the phytochemical profiles and antioxidant capacity of *G. stenostachya* and *W. macrochlamys* under controlled temperature and irrigation-frequency treatments through the identification of secondary metabolites by gas chromatography–mass spectrometry (GC-MS), the determination of total phenolic content using the Folin–Ciocalteu assay, and the evaluation of antioxidant capacity using the DPPH and ABTS assays, in order to determine changes in their chemical composition and antioxidant capacity associated with both types of stress. In this way, the study provides novel information on the plasticity of secondary metabolism in two Bromeliaceae species that have been scarcely studied from a phytochemical perspective and on their responses to environmental factors relevant in the context of climate change.

## 2. Results

### 2.1. Phenolic and Antioxidant Capacity to Temperature and Irrigation-Frequency Treatments

#### 2.1.1. Total Phenolic Content

This subsection presents the total phenolic content (TPC) and antioxidant capacity by the ABTS and DPPH assays following the temperature treatments: T1, T2, and T3 corresponded to 23 °C, 26 °C, and 29 °C, respectively. Irrigation-frequency treatments corresponded to 24 h (RH1), 48 h (RH2), and 72 h (RH3).

In temperature treatments, *G. stenostachya*, TPC decreased from 380.85 ± 5.08 mg GAE 100 g^−1^ at 23 °C to 274.81 ± 3.75 mg GAE 100 g^−1^ at 26 °C, and subsequently increased to 430.97 ± 5.13 mg GAE 100 g^−1^ at 29 °C. In *W. macrochlamys*, a continuous decrease was observed, from 711.45 ± 7.77 mg GAE 100 g^−1^ at 23 °C to 582.43 ± 13.72 and 508.37 ± 14.50 mg GAE 100 g^−1^ at 26 and 29 °C, respectively. *W. macrochlamys* exhibited higher TPC values throughout the entire temperature gradient (Figure 1).

Under irrigation-frequency treatments, in *G. stenostachya*, RH2 showed the lowest TPC (396.31 ± 2.04 mg GAE 100 g^−1^), followed by RH1 (424.86 ± 4.26 mg GAE 100 g^−1^), whereas the highest value was recorded in RH3 (555.05 ± 2.64 mg GAE 100 g^−1^). In *W. macrochlamys*, the lowest value was observed in RH1 (409.06 ± 12.76 mg GAE 100 g^−1^), the highest in RH2 (756.25 ± 3.33 mg GAE 100 g^−1^), and an intermediate value in RH3 (483.28 ± 11.05 mg GAE 100 g^−1^) (Figure 2).

#### 2.1.2. Antioxidant Capacity by ABTS Assay

In temperature treatments, *G. stenostachya*, antioxidant capacity decreased from 6127.44 ± 72.15 µmol TE 100 g^−1^ at 23 °C to 5269.38 ± 334.34 µmol TE 100 g^−1^ at 26 °C and remained at a similar level at 29 °C (5329.37 ± 540.15 µmol TE 100 g^−1^). In *W. macrochlamys*, a continuous decrease was observed, from 11,489.88 ± 44.81 µmol TE 100 g^−1^ at 23 °C to 8436.89 ± 340.17 and 7118.81 ± 244.39 µmol TE 100 g^−1^ at 26 and 29 °C, respectively. *W. macrochlamys* maintained higher values throughout the entire temperature gradient (Figure 3).

Under the irrigation-frequency treatments, in *G. stenostachya*, RH2 showed the lowest antioxidant activity (7095.39 ± 252.63 µmol TE 100 g^−1^), followed by RH1 (8209.58 ± 181.52 µmol TE 100 g^−1^), whereas the highest value was recorded in RH3 (9408.63 ± 597.84 µmol TE 100 g^−1^). In *W. macrochlamys*, RH2 resulted in the highest antioxidant activity (10,494.51 ± 201.13 µmol TE 100 g^−1^), whereas RH1 (6209.89 ± 264.08 µmol TE 100 g^−1^) and RH3 (6877.79 ± 398.19 µmol TE 100 g^−1^) (Figure 4).

#### 2.1.3. Antioxidant Capacity by DPPH Assay

In temperature treatments, *G. stenostachya*, the value decreased from 1895.30 ± 22.74 µmol TE 100 g^−1^ at 23 °C to 1290.01 ± 46.04 µmol TE 100 g^−1^ at 26 °C, and subsequently increased to 1630.64 ± 10.96 µmol TE 100 g^−1^ at 29 °C. In *W. macrochlamys*, a continuous decrease was observed, from 2847.83 ± 56.93 µmol TE 100 g^−1^ at 23 °C to 2656.17 ± 27.35 and 2554.97 ± 39.16 µmol TE 100 g^−1^ at 26 and 29 °C, respectively. *W. macrochlamys* maintained higher values throughout the entire temperature gradient (Figure 5).

Under the irrigation-frequency treatments, *G. stenostachya*, RH2 showed the lowest value (1753.12 ± 27.69 µmol TE 100 g^−1^), compared to RH1 (2255.04 ± 14.04 µmol TE 100 g^−1^) and RH3 (2150.73 ± 73.97 µmol TE 100 g^−1^). In *W. macrochlamys*, the highest antioxidant activity was recorded in RH2 (2921.60 ± 33.61 µmol TE 100 g^−1^), compared to the data for RH1 (2557.51 ± 65.39 µmol TE 100 g^−1^) and RH3 (2560.77 ± 201.22 µmol TE 100 g^−1^) (Figure 6).

### 2.2. Phytochemical Profile by GC-MS

Table 1 shows variations in the number and composition of tentative compounds detected by GC-MS in *G. stenostachya* and *W. macrochlamys* under the different temperature treatments (representative chromatograms are presented in Supplementary Figures S1–S7). In *G. stenostachya*, seven compounds were identified at both 23 and 26 °C, whereas at 29 °C the number increased to nine, making this the condition with the highest number of detected compounds. n-Hexadecanoic acid, Butanoic acid, Benzeneacetaldehyde, and (+)-2-Bornanone were present at all three temperatures. Treatment-specific variations were also observed: Limonene was detected only at 23 °C and Pentanoic acid only at 26 °C, whereas α-Campholenal, 5-Ethenyl-2-methoxyphenol, and Neophytadiene were identified exclusively at 29 °C. Fenchone was present at 23 and 29 °C, whereas 2(4H)-Benzofuranone, 5,6,7,7a-tetrahydro-4,4,7a-trimethyl- was detected at 26 and 29 °C.

In *W. macrochlamys*, the number of detected compounds decreased from eight at 23 °C to six at 26 °C and remained at six at 29 °C. As in *G. stenostachya*, n-Hexadecanoic acid, Butanoic acid, Benzeneacetaldehyde, and (+)-2-Bornanone were detected under all three thermal conditions. Some compounds showed a more specific distribution, such as 4-Hydroxybenzaldehyde and (9Z,12Z,15Z)-Octadeca-9,12,15-trien-1-ol, which were observed only at 23 °C, whereas Limonene was present at 26 and 29 °C. 2(4H)-Benzofuranone, 5,6,7,7a-tetrahydro-4,4,7a-trimethyl- was detected at 23 and 26 °C, whereas 2,3-Dihydrobenzofuran was present at 23 and 29 °C, demonstrating differences in chemical composition among the evaluated temperatures.

Under irrigation-frequency treatments (representative chromatograms are presented in Supplementary Figures S8–S13), differences were also observed in the profiles obtained for both species throughout the evaluated period. In *G. stenostachya*, six compounds were identified at 24 h, four at 48 h, and seven chromatographic records were obtained at 72 h. Benzeneacetaldehyde, (+)-2-Bornanone, and n-Hexadecanoic acid were recurrent at all three time points, whereas Limonene was detected only at 24 h. At 72 h, a greater number of compounds were again observed, including Neophytadiene, Vanillin, and 2(4H)-Benzofuranone, 5,6,7,7a-tetrahydro-4,4,7a-trimethyl-, which were not recorded at 24 or 48 h.

In *W. macrochlamys*, a different trend was observed under irrigation-frequency treatments, with a progressive increase in the number of detected compounds, from three at 24 h to four at 48 h and five at 72 h. Butanoic acid, Benzeneacetaldehyde, and (+)-2-Bornanone were present throughout the entire evaluation period, whereas Neophytadiene was detected at 48 h, and 2,3-Dihydrobenzofuran and 5-Ethenyl-2-methoxyphenol appeared at 72 h. Overall, (+)-2-Bornanone and Benzeneacetaldehyde were the only compounds detected under all temperature and irrigation-frequency treatments, conditions evaluated in both species, whereas the remaining compounds showed distributions associated with species and experimental condition.

## 3. Discussion

The two species exhibited different phytochemical responses to increasing temperature. *W. macrochlamys* showed higher total phenolic content (TPC) and greater antioxidant capacity through the ABTS and DPPH assays throughout the temperature gradient; however, all three parameters progressively decreased from 23 to 29 °C. In contrast, *G. stenostachya* exhibited a nonlinear response, characterized by a decrease in TPC at 26 °C followed by a significant increase at 29 °C. These differences are consistent with the influence of environmental factors and species-specific characteristics on the synthesis and accumulation of secondary metabolites [33]. Likewise, the response of phenolic compounds to thermal stress has been reported to depend on the species, stress intensity, and the ability of the plant to respond to these conditions [34].

The increase in TPC observed in *G. stenostachya* at 29 °C indicated changes in secondary metabolism in response to the highest temperature used in this experiment. Phenolic compounds participate in plant antioxidant mechanisms through, among other processes, their ability to transfer electrons and neutralize reactive species [35]. However, the increase in TPC was not accompanied by a proportional increase in antioxidant capacity, since ABTS values remained relatively stable between 26 and 29 °C, whereas DPPH showed only a slight increase. This indicates that the antioxidant capacity cannot be explained solely by total phenolic content. Independently, GC-MS analysis revealed the highest number of detectable compounds in *G. stenostachya* at 29 °C, including α-campholenal, 5-ethenyl-2-methoxyphenol, and neophytadiene, which were not observed at the other temperatures. However, the present data does not allow these compositional changes to be directly associated with the antioxidant measurements.

In *W. macrochlamys*, TPC and antioxidant capacity progressively decreased with increasing temperature. Additionally, GC-MS profiling showed a reduction in the number of detectable compounds, from eight at 23 °C to six at both 26 and 29 °C. These parallel trends should not be interpreted as evidence of a direct relationship. Despite maintaining higher values than *G. stenostachya*, the response of this species progressively decreased with increasing temperature. However, the TPC recorded in both species was higher than that reported for *Nidularium procerum* [36]. Nevertheless, differences among studies may be influenced by the solvent and extraction conditions, as well as by the procedure used to determine phenolic compounds. Such variations have been identified as factors affecting comparisons of phytochemical results among studies. An association between phenolic content and antioxidant capacity has also been reported in other Bromeliaceae [37]. Overall, the results demonstrate distinct phytochemical responses of the two species to increasing temperature.

Previous experiments conducted with these same species showed that a temperature increase from 23 to 26 and 29 °C reduced germination more markedly in *G. stenostachya*, while *W. macrochlamys* maintained comparatively higher germination rates at elevated temperatures, suggesting differences in their ability to tolerate temperature variation [13]. Although germination and leaf phytochemical responses represent distinct physiological processes, these findings provide evidence that both species do not respond uniformly to temperature changes at different stages of their development. *W. macrochlamys* maintained higher concentrations of phenolic compounds and overall antioxidant capacity across the different temperatures, despite experiencing a progressive decline as temperature increased, whereas *G. stenostachya* exhibited a nonlinear response, including an increase in total phenolic content (TPC) and greater chemical diversity at 29 °C.

Because epiphytic bromeliads grow without direct access to soil water, their water supply relies largely on intermittent atmospheric inputs such as rainfall, fog, and dew. This ecological constraint has favored structural and physiological traits that enhance water interception and temporary storage [12]. Accordingly, variation in irrigation frequency may influence physiological and metabolic responses in these plants, although these effects may differ between species.

Irrigation-frequency treatments produced an even more contrasting pattern between the two species. In *G. stenostachya*, 48 h was associated with the lowest TPC, ABTS, and DPPH values, whereas a marked slight increase was observed at 72 h. In *W. macrochlamys*, the opposite pattern occurred, with the highest values for all three parameters recorded at 48 h, followed by a subsequent decrease. This pattern explains the significant interaction observed between species and treatment and further indicates that the phytochemical response to water stress may depend both on species and irrigation-frequency treatments.

The simultaneous increase in TPC and antioxidant capacity in *W. macrochlamys* at 48 h revealed a more intense phytochemical response to irrigation-frequency treatments. Drought can modify the biosynthesis of secondary metabolites and regulate pathways related to the production of phenolic compounds, including the phenylpropanoid pathway [38]. In Bromeliaceae, [39] also described antioxidant mechanisms associated with secondary metabolites in *P. burchellii* under water deficit. In contrast, the transient decrease observed in *G. stenostachya* showed changes in resource allocation between primary and secondary metabolism during stress, a response previously described in plants exposed to environmental constraints [40]. However, these mechanisms were not directly assessed in the present study and should therefore be considered plausible explanations for the observed patterns.

At 72 h, *G. stenostachya* exhibited its most pronounced phytochemical response, whereas *W. macrochlamys* showed a decrease relative to the maximum recorded at 48 h. The intensity and duration of drought can progressively modify antioxidant systems and secondary metabolite production [41,42]. In this regard, at 48 h the results suggest a more intense response in *W. macrochlamys* and a lower intensity response in *G. stenostachya*, without necessarily implying direct differences in physiological tolerance. Although ABTS and DPPH reflected some of these trends, the two assays did not show identical responses: according to the ABTS assay, *G. stenostachya* showed higher values at 24 and 72 h, whereas according to the DPPH assay, *W. macrochlamys* maintained higher values at all three time points. This difference reinforces the importance of considering both assays as complementary measures of antioxidant capacity.

The antioxidant capacity observed in these species is consistent with previous reports for other Bromeliaceae, including *Bromelia laciniosa* [43], *Encholirium spectabile* [37], *Bromelia balansae* [44], and *Bromelia antiacantha* [45]. However, the patterns recorded in the present study show that this characteristic can vary considerably depending on the species and environmental conditions, an aspect that is particularly relevant for understanding the biochemical responses of bromeliads to abiotic stress.

In Bromeliaceae, water deficit has been shown to modify antioxidant metabolism in *Guzmania monostachia*, [46]. Likewise, increased antioxidant activity during the dry season has been reported in the bromeliads *Tillandsia brachycaulos* and *Bromelia karatas*, where plants were simultaneously exposed to reduced water availability and higher irradiance [47]. This context may explain why *W. macrochlamys* and *G. stenostachya* displayed different response maxima under the irrigation-frequency treatments.

Regarding the GC-MS analysis, temperature and irrigation-frequency treatments were accompanied by qualitative variations in the chemical composition of both species. Environmental conditions can modify the production and accumulation of plant metabolites [33], and terpenoids, phenolic compounds, and lipid derivatives have been identified in other Bromeliaceae, including *Greigia sphacelata* [48] and *Bromelia laciniosa* [49]. In the present study, the number of compounds detected in *G. stenostachya* increased from seven at both 23 and 26 °C to nine at 29 °C, whereas in *W. macrochlamys* it decreased from eight to six. Under irrigation-frequency treatments, *G. stenostachya* showed six, four, and seven compounds at 24, 48, and 72 h, respectively, whereas *W. macrochlamys* showed a progressive increase from three to five compounds. These patterns support variation in the chemical profile that depends on both species and stress condition.

(+)-2-Bornanone and Benzeneacetaldehyde were the only compounds detected under all conditions evaluated in both species, whereas other compounds showed a more specific distribution. Limonene, Neophytadiene, and 5-Ethenyl-2-methoxyphenol occurred under particular conditions depending on the species and treatment applied. These differences demonstrate qualitative variation in the chemical profile associated with the experimental conditions. The presence of terpenoids is consistent with studies conducted in *Tillandsia*, in which a broad diversity of monoterpenes and other volatile compounds has been reported [50]. Likewise, the detection of phenolic compounds is consistent with the phytochemical diversity described in *Neoglaziovia variegata* [33].

The GC-MS analysis characterized the GC-amenable fraction of the hydroethanolic extracts rather than providing a comprehensive phytochemical profile. Because the extracts were analyzed without derivatization, coverage of polar, non-volatile, and thermolabile metabolites was limited, including many phenolic acids and flavonoids that may contribute to the measured TPC and antioxidant capacity. Consequently, the tentatively annotated compounds cannot fully account for these measurements. In addition, drying can alter phenolic composition and antioxidant activity [51]. Oven drying at 60 °C for 48 h may therefore have affected the chemical composition of the extracts, including possible losses of volatile compounds and the degradation of thermolabile constituents. Future studies should incorporate complementary Liquid Chromatography (LC)-based profiling to obtain broader coverage of these metabolites and further investigate their potential contributions to antioxidant capacity.

Several tentative compounds detected by GC-MS in the present study have previously been associated with antioxidant properties. Among them, Vanillin, Limonene, n-Hexadecanoic acid. Their occurrence in the extracts therefore suggests that these metabolites could contribute, together with other constituents, to the overall antioxidant capacity by ABTS and DPPH. However, their individual contribution cannot be established from the present data because these compounds were not evaluated separately for antioxidant activity. Moreover, the antioxidant response of the extracts likely reflects the combined effects of multiple metabolites, including polar phenolic compounds that may not have been adequately represented by the GC-MS analysis. Therefore, the antioxidant activities previously reported for these GC-MS-detected compounds should be considered as potential contributions rather than evidence of a direct relationship with the ABTS or DPPH values observed in this study.

The detected compounds can be grouped into classes with distinct metabolic origins. Monoterpenes are primarily biosynthesized via the plastidial MEP pathway, whereas phenylpropanoids/benzenoids derive mainly from L-phenylalanine originating from the shikimate pathway [52,53]. The detection of 2(4H)-benzofuranone, 5,6,7,7a-tetrahydro-4,4,7a-trimethyl- is consistent with carotenoid oxidative cleavage, whereas the occurrence of short-chain fatty acids may reflect changes in lipid-associated catabolic processes [54,55,56]. In *G. stenostachya*, the greater number of compounds detected at 29 °C resulted mainly from the appearance of α-campholenal, 5-ethenyl-2-methoxyphenol, and neophytadiene, indicating a broader chemical response rather than a uniform increase in a single metabolic pathway. Therefore, the higher chemical diversity observed at 29 °C may reflect stress-induced metabolic reorganization involving different biosynthetic and catabolic processes. The lack of a proportional increase in ABTS and DPPH further indicates that the appearance of additional GC-MS-detectable compounds was not necessarily accompanied by a corresponding increase in overall antioxidant capacity.

The non-linear variation in total phenolic content observed in *G. stenostachya* suggests a stress-intensity-dependent reorganization of phenolic metabolism. Plant responses to stress can be highly dose-dependent, and mild and severe stress conditions may differentially affect defense metabolism. Low-intensity stress has been associated with the stimulation of non-enzymatic antioxidants, including phenolic compounds, whereas stronger or prolonged stress may produce different or even inhibitory responses [57]. Moreover, different physiological and biochemical endpoints do not necessarily respond synchronously to the same stress intensity. Such hormetic trade-offs have been described when the stimulation of one response is accompanied by inhibition or no change in another [57], providing a possible framework for understanding the lack of parallel variation between total phenolic content and antioxidant capacity observed here.

Benzeneacetaldehyde was the only compound, together with (+)-2-bornanone, detected under all conditions evaluated in both species. Benzeneacetaldehyde is a phenylalanine-derived benzenoid; in plants, its biosynthesis can involve the decarboxylation of phenylalanine to phenethylamine, followed by its conversion to phenylacetaldehyde [58]. Phenylalanine is also the entry substrate of the phenylpropanoid pathway, where its conversion to trans-cinnamic acid by phenylalanine ammonia-lyase initiates the biosynthesis of numerous phenolic compounds [59,60]. Thus, although the GC-MS profile and total phenolic content represent analytically distinct measurements, they provide complementary information on different branches of secondary metabolism that may share common upstream precursors. Accordingly, changes in benzeneacetaldehyde abundance should not be expected to parallel changes in total phenolic content directly, because metabolic flux from phenylalanine can be differentially distributed among volatile benzenoid and phenylpropanoid-derived metabolites.

Although the evaluation of biotechnological or nutraceutical applications was not a direct objective of this study, the presence of metabolites for which antioxidant, antimicrobial, and other biological activities have previously been reported suggests that both species may be of interest for future research. Nevertheless, the variation observed among species and treatments indicates that this potential could be influenced by environmental factors and by the specific metabolic response of each bromeliad species.

Overall, the integration of TPC, ABTS, DPPH, and GC-MS analyses addresses part of the existing knowledge gap regarding *G. stenostachya* and *W. macrochlamys*. The results show that both species modify their phytochemical composition and antioxidant capacity in response to temperature and irrigation-frequency treatments, although they follow different response patterns. *W. macrochlamys* maintained greater antioxidant capacity throughout the temperature gradient, although it progressively decreased with increasing temperature, whereas *G. stenostachya* exhibited a nonlinear response accompanied by greater chemical diversity at 29 °C. Under irrigation-frequency treatments, *W. macrochlamys* showed a more pronounced response at 48 h, whereas *G. stenostachya* showed its strongest response at 72 h. Independently, GC-MS profiling showed greater detectable chemical diversity in *G. stenostachya* at 29 °C. These datasets should be regarded as complementary rather than directly correlated.

These findings provide initial evidence of phytochemical plasticity dependent on both species and temperature, and irrigation-frequency treatments, expanding current knowledge of two Bromeliaceae species that have thus far been scarcely studied from this perspective.

## 4. Materials and Methods

### 4.1. Plant Material

Individuals of *W. macrochlamys* and *G. stenostachya* were collected from the Fortuna Forest Reserve, located in Chiriquí Province, Panama (8°43′20″ N, 82°14′15″ W).

The plants were identified using the Bromeliaceae family key from the Manual de Plantas de Costa Rica. The specimens are deposited in the UCH Herbarium at the Universidad Autónoma de Chiriquí under voucher UCH-C007-08-2025.

All collected plants were at the adult stage, and individuals of comparable size were selected to minimize variability associated with plant development and dimensions. Prior to the start of the experimental treatments, the plants underwent a one-week acclimatization period.

Subsequently, the plants were transported to the laboratory for the application of experimental treatments. Five individuals per species were used for each treatment (temperature and irrigation-frequency treatments). All the plants were placed in pots with tree bark as substrate. Following exposure to the treatments in climate chambers, leaves were collected for chemical analyses.

### 4.2. Climate Chambers and Experimental Conditions

Three experimental independent climate chambers were constructed to allow precise control of temperature, relative humidity, and irrigation regime. All chambers were programmed with a 12 h light photoperiod using blue led light 2527 lumens (AquaLux Plants Aquarium Light Nicrew,; Nicrew, Hangzhou, China). Humidifiers were also installed inside each chamber to maintain relative humidity between 95 and 98%, together with heating elements (150 W ceramic heater, Repti Home, Changzhou, China) and ventilation systems to ensure homogeneous thermal conditions throughout the experimental space.

For the temperature treatments experiment, Chamber 1 was maintained at a constant temperature of 23 °C, considered the baseline or control condition; Chamber 2 was set at 26 °C, representing a moderate temperature increase; and Chamber 3 was maintained at 29 °C, simulating more pronounced warming conditions for 60 days. This experimental design allowed the reproduction of a range of thermal conditions comparable to climate change scenarios projected for tropical environments. After the 60-day period, samples were collected, and a composite sample was prepared for subsequent analysis.

For the irrigation-frequency treatments, all three chambers were maintained at a constant temperature of 23 °C; only the irrigation frequency was varied to ensure that any observed responses could be attributed exclusively to the irrigation-frequency treatment. In this experiment, the treatments differed in spry-irrigation frequency. Plants in the first chamber were irrigated every 24 h (RH1), those in the second chamber every 48 h (RH2), and those in the third chamber every 72 h (RH3). At each scheduled irrigation, each plant received 20 mL of water by spraying. The experiment lasted 60 days; at the end of this period, the plants were collected, and a composite sample was prepared for subsequent analysis.

### 4.3. Extraction Method

Leaves from five individuals of each species and treatment were combined to obtain a composite sample, due to the limited amount of sample material. The plant material was subsequently cleaned, dried at 60 °C for 48 h in an oven Heratherm OMS100 (Thermo Scientific, Langenselbold, Germany), ground, sieved, and stored in plastic bags in the dark until analysis. Extracts were obtained by ultrasound-assisted extraction following the methodology described by [61], with modifications. Briefly, 0.5 g of dried sample was weighed, 10 mL of a 60% hydroethanolic solution was added, and the mixture was sonicated for 15 min in an ultrasonic bath Elma P30H (Singen, Germany) at 60 ± 3 °C. The samples were subsequently centrifuged Joanlab LC-500 (Shenyang, Huzhou, China) at 2146 *g* for 3 min. The resulting extracts were filtered through 0.22 µm nylon syringe filters (Biomed Scientific Ltd., Shenzhen, China) and stored under refrigerated conditions until analysis.

### 4.4. Determination of Total Phenolic Content

Total phenolic content was determined using three analytical replicates per composite sample and the Folin–Ciocalteu reagent method, following [62]. A 500 µL aliquot of hydroethanolic extract was transferred into a test tube, and 2500 µL of Folin–Ciocalteu reagent (0.2 mol L^−1^) (St Louis, MO, USA) was added. The mixture was allowed to stand for 5 min, after which 2 mL of 7.5% sodium carbonate (St Louis, MO, USA) solution was added. The mixture was vortexed and incubated in the dark at room temperature for 120 min. Absorbance was measured at 748 nm using a spectrophotometer UV-1800 spectrophotometer (BRT, Shenyang, Liaoning, China). Gallic acid (St Louis, MO, USA) was used as the reference standard at concentrations ranging from 0 to 10 µg mL^−1^. Total phenolic content was expressed as milligrams of gallic acid equivalents per 100 g of dry mass (mg GAE 100 g^−1^ dry mass).

### 4.5. Determination of Antioxidant Capacity Using the ABTS Assay

The ABTS assay was performed using three analytical replicates per composite sample according to the methodology described by [61]. The ABTS^+^ radical was generated by mixing ABTS (7 mM) with potassium persulfate (2.45 mM), followed by incubation in the dark for 16 h at 25 °C. The ABTS^+^ radical solution was adjusted to an absorbance of 0.77 ± 0.02 at 754 nm and mixed with 100 µL of the extract. The mixture was then incubated in the dark for 6 min. After incubation, absorbance was measured at 754 nm using a spectrophotometer (UV-1800 spectrophotometer, BRT, Shenyang, Liaoning, China). Trolox was used as the reference standard at concentrations ranging from 40 to 320 µM, and the results were expressed as micromoles of Trolox equivalents per gram of dry mass (µmol TE 100 g^−1^).

### 4.6. Determination of Antioxidant Capacity Using the DPPH Assay

The DPPH assay was performed using three analytical replicates per composite sample according to [61], using a methanolic (Tedia, Fairfield, CT, USA) DPPH (St Louis, MO, USA) solution adjusted to an absorbance of 0.70 ± 0.02 at 515 nm. A 100 µL aliquot of the diluted extract was mixed with the DPPH solution and incubated in the dark for 30 min. After the incubation period, absorbance was measured at 515 nm using a spectrophotometer (UV-1800 spectrophotometer, BRT, Shenyang, Liaoning, China). Trolox (St Louis, MO, USA) was used as the reference standard at concentrations ranging from 60 to 540 µM, and the results were expressed as micromoles of Trolox equivalents per gram of dry mass (µmol TE 100 g^−1^).

### 4.7. GC-MS Characterization

Hydroethanolic extracts of *W. macrochlamys* and *G. stenostachya* from each treatment were analyzed by gas chromatography–mass spectrometry (GC-MS) using an Agilent 8890 GC system (Agilent Technologies, Santa Clara, CA, USA) coupled to an Agilent 5977C single-quadrupole mass selective detector (MSD) (Agilent Technologies, Santa Clara, CA, USA) equipped with an extractor-type electron ionization source (EI-XTR). Chromatographic separation was performed using an HP-5MS capillary column (5% phenyl–95% dimethylpolysiloxane; 30 m × 0.25 mm i.d. × 0.25 µm film thickness). Helium was used as the carrier gas at a constant flow rate of 1.0 mL min^−1^. Samples (1 µL) were injected in split mode (5:1) at an injector temperature of 250 °C. The oven temperature program started at 50 °C and was held for 2 min, then increased to 100 °C at a rate of 4 °C min^−1^ and held for 3 min. The temperature was subsequently increased to 200 °C at 5 °C min^−1^ and held for 5 min, followed by an increase to 280 °C at 5 °C min^−1^ with a 5 min hold, and finally increased to 300 °C at 5 °C min-1 and held for an additional 5 min.

The mass spectrometer was operated in electron ionization (EI) mode at an ionization energy of 70 eV. The transfer line temperature was maintained at 270 °C, while the ion source and quadrupole temperatures were set at 230 and 150 °C, respectively. Data were acquired in scan mode over an *m*/*z* range of 35–500. Samples were injected directly without additional dilution or prior derivatization.

### 4.8. GC-MS Data Processing and Tentative Compound Annotation

Each extract was injected in duplicate. Post-acquisition data processing was performed using Agilent MassHunter Unknowns Analysis software version 12.1, with identical parameters applied to all samples.

Components were detected by spectral deconvolution using a detection threshold of 0.05% relative to the height of the most intense chromatographic peak. The deconvolution parameters were as follows: retention time window size factors of 25, 50, 100, and 200; an extraction window with left and right delta *m*/*z* values of 0.3 and 0.7 AMU, respectively, using integer *m*/*z* values; a sharpness threshold of 25%; a minimum of three ion peaks per component; a maximum of ten stored ion-peak shapes; normal sensitivity; and a subregion retention time window of 3. Blank subtraction was applied.

Compounds were annotated by comparing the deconvoluted spectra against the NIST 23 mass spectral library. Spectral searches were performed using a normal presearch, with score adjustment enabled and a pure weight factor of 0.7 for forward–reverse searching. A minimum match factor of 85 and a minimum mass-to-charge ratio of 30 were applied as initial criteria for tentative annotation. Library match scores for the compounds reported in Table 1 ranged from 85.0% to 99.8%.

Retention index information provided additional support for compound annotation. Experimental retention indices were calculated using a C8–C40 n-alkane series analyzed under the same conditions.

Because authentic standards were not analyzed in the same laboratory for final confirmation, all compounds are reported as tentatively annotated, with supporting retention index information, rather than as definitively identified structures [63,64].

### 4.9. Statistical Analysis

Statistical analyses were performed using R, version 4.6.0 (R Core Team, R Foundation for Statistical Computing, Vienna, Austria). For the experiments for both the temperature and irrigation-frequency treatments, total phenolic content (TPC) and antioxidant capacity measured using the ABTS and DPPH assays were summarized using descriptive statistics. Results were expressed as the mean ± standard deviation (SD) of three technical replicates obtained from a single composite sample consisting of leaves pooled from five plants per species and treatment.

## 5. Conclusions

To our knowledge, this study is among the first to combine GC-MS profiling, total phenolic content (TPC), and ABTS and DPPH assays in a comparative evaluation of *G. stenostachya* and *W. macrochlamys* under temperature and irrigation-frequency treatments. By examining these complementary chemical and antioxidant measurements under defined experimental conditions, the study provides an initial experimental reference for two Bromeliaceae species whose phytochemical characteristics remain scarcely explored.

In the analyzed composite samples, *W. macrochlamys* exhibited its highest mean TPC, ABTS, and DPPH values at 23 °C: 711.45 mg GAE 100 g^−1^, 11,489.88 µmol TE 100 g^−1^, and 2847.83 µmol TE 100 g^−1^, respectively. In *G. stenostachya*, the highest mean TPC occurred at 29 °C (430.97 mg GAE 100 g^−1^), whereas the highest ABTS and DPPH values occurred at 23 °C (6127.44 and 1895.30 µmol TE 100 g^−1^, respectively). Thus, the temperature associated with the highest TPC did not consistently coincide with that associated with the highest antioxidant capacity.

Under the irrigation-frequency treatments, *W. macrochlamys* exhibited its highest mean TPC, ABTS, and DPPH values with irrigation every 48 h: 756.25 mg GAE 100 g^−1^, 10,494.51 µmol TE 100 g^−1^, and 2921.60 µmol TE 100 g^−1^, respectively. In *G. stenostachya*, the highest mean TPC and ABTS values occurred with irrigation every 72 h (555.05 mg GAE 100 g^−1^ and 9408.63 µmol TE 100 g^−1^, respectively), whereas the highest DPPH value occurred with irrigation every 24 h (2255.44 µmol TE 100 g^−1^). These observations indicate that the irrigation frequency associated with the highest measured value differed between species and, in *G. stenostachya*, among analytical endpoints.

GC-MS profiling provided complementary evidence of qualitative differences among the extracts. In *G. stenostachya*, seven compounds were detected at both 23 and 26 °C and nine at 29 °C, whereas the corresponding counts in *W. macrochlamys* were eight, six, and six, respectively. With irrigation every 24, 48, and 72 h, *G. stenostachya* showed six, four, and seven detected compounds, respectively; counts in *W. macrochlamys* ranged from three to five across the irrigation-frequency treatments. The larger number of compounds detected in *G. stenostachya* at 29 °C did not coincide with its highest ABTS or DPPH values. These findings show the value of combining chemical profiling with antioxidant assays, although the contributions of individual compounds to the measured antioxidant capacity remain undetermined.

Together, these findings help address a specific gap in the phytochemical characterization of *G. stenostachya* and *W. macrochlamys* and provide a basis for selecting species and experimental conditions for further investigation. Interpretation of the results is limited by the use of composite samples without the analysis of independent biological replicates and by the analytical coverage of GC-MS without derivatization, particularly for polar, non-volatile, and thermolabile constituents. Future studies should prioritize the analysis of independent biological replicates, the confirmation and quantification of tentatively annotated compounds, and complementary liquid chromatography (LC)-based profiling. Further biological activity assessments are needed to evaluate potential biotechnological or nutraceutical applications. The comparative data generated here provide a starting point for investigating the chemical constituents and environmental responses of these understudied bromeliads.

## Figures and Tables

**Figure 1:**
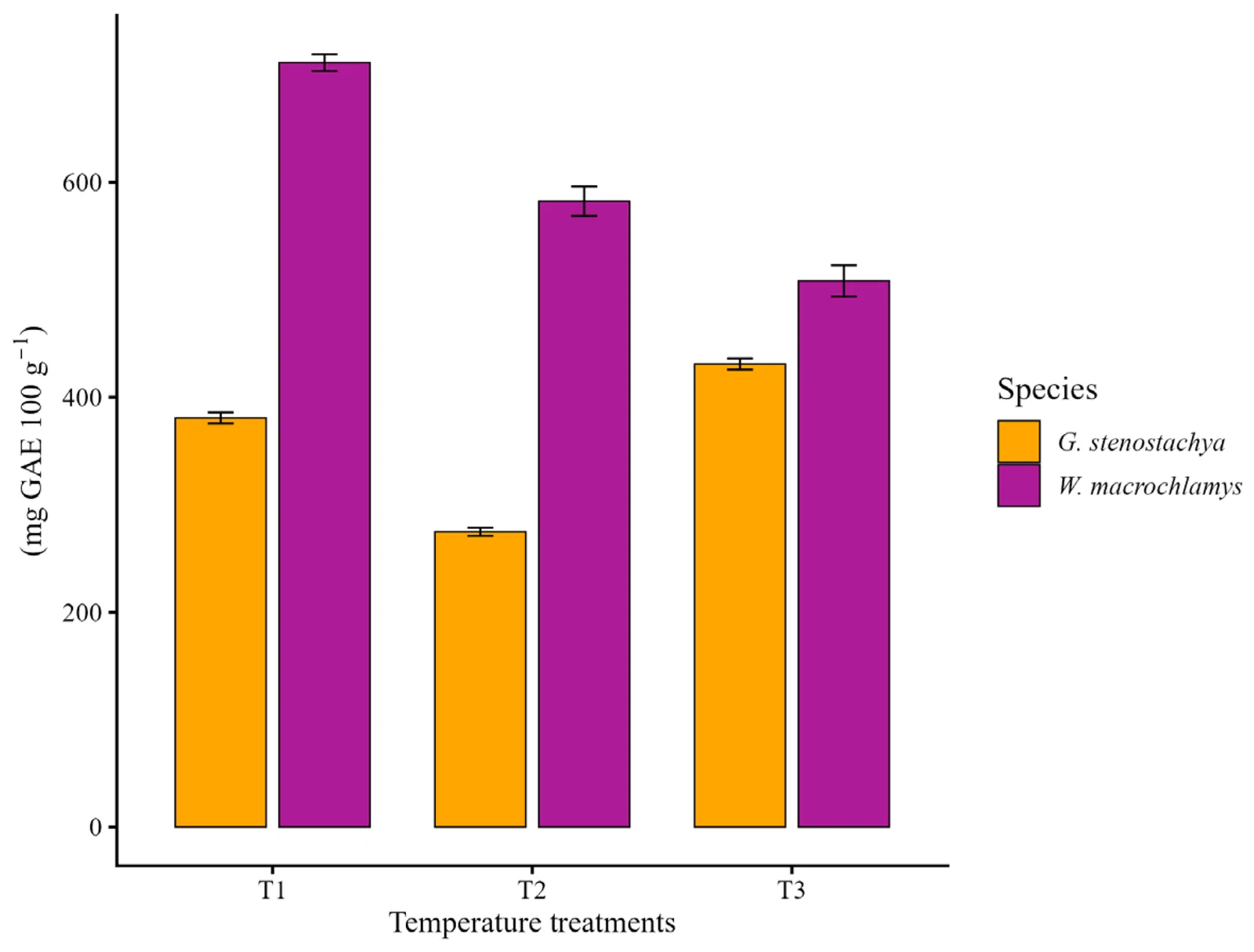
Effect of temperature treatments on TPC in *G. stenostachya* and *W. macrochlamys*. Values are expressed as mean ± SD.

**Figure 2:**
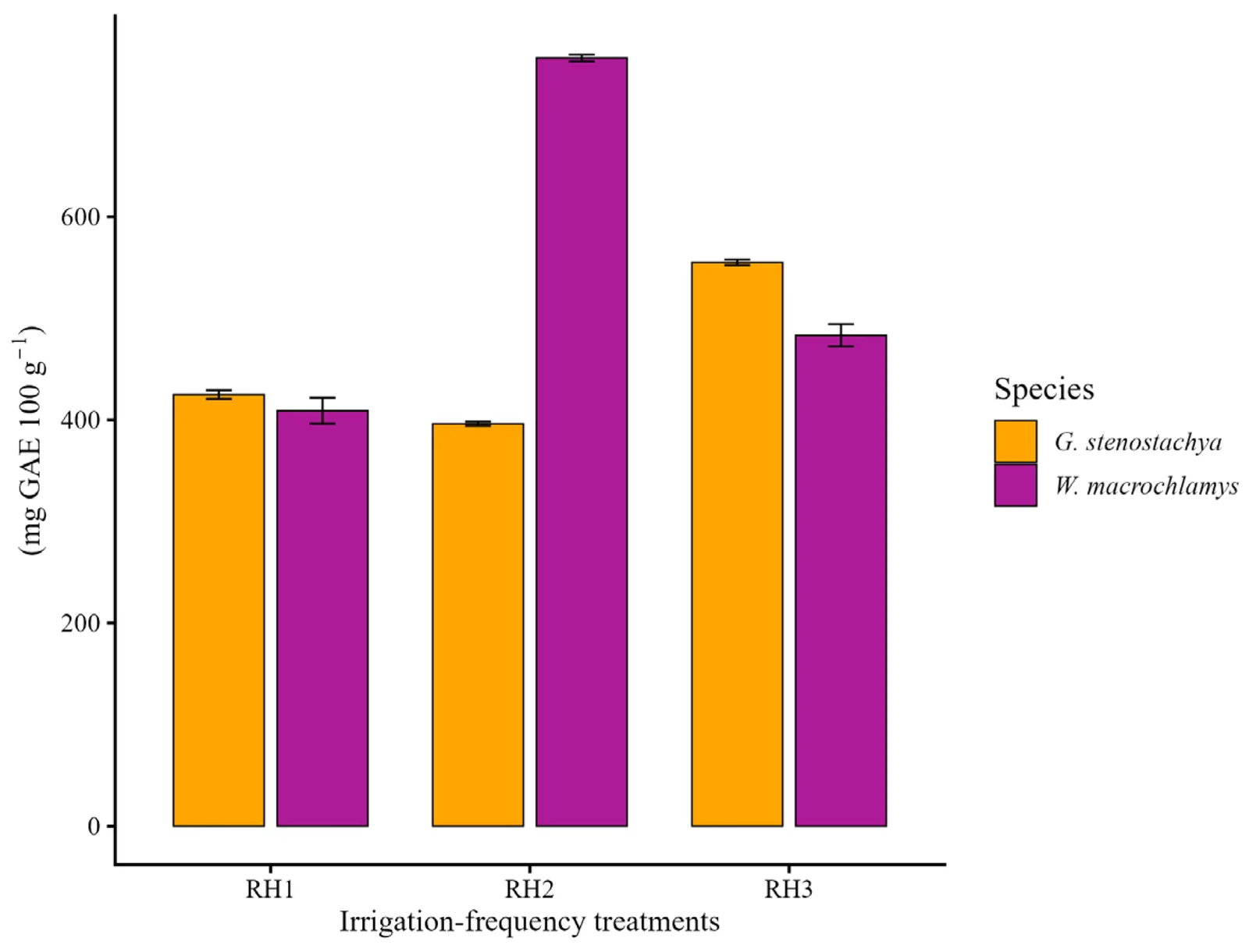
Effect of irrigation-frequency treatments on TPC in *G. stenostachya* and *W. macrochlamys*. Values are expressed as mean ± SD.

**Figure 3:**
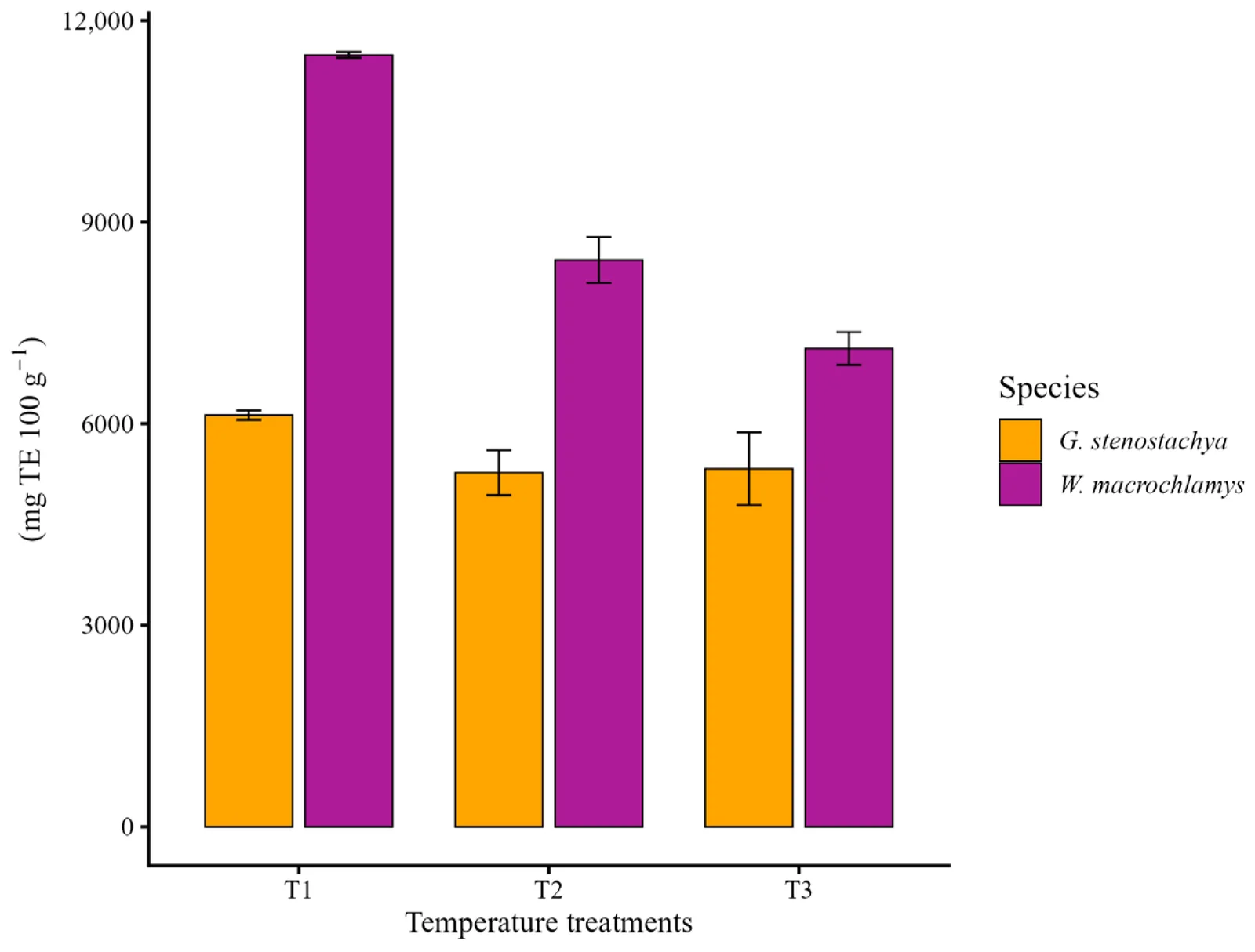
Effect of temperature treatments on antioxidant capacity by ABTS assay in *G. stenostachya* and *W. macrochlamys.* Values are expressed as mean ± SD.

**Figure 4:**
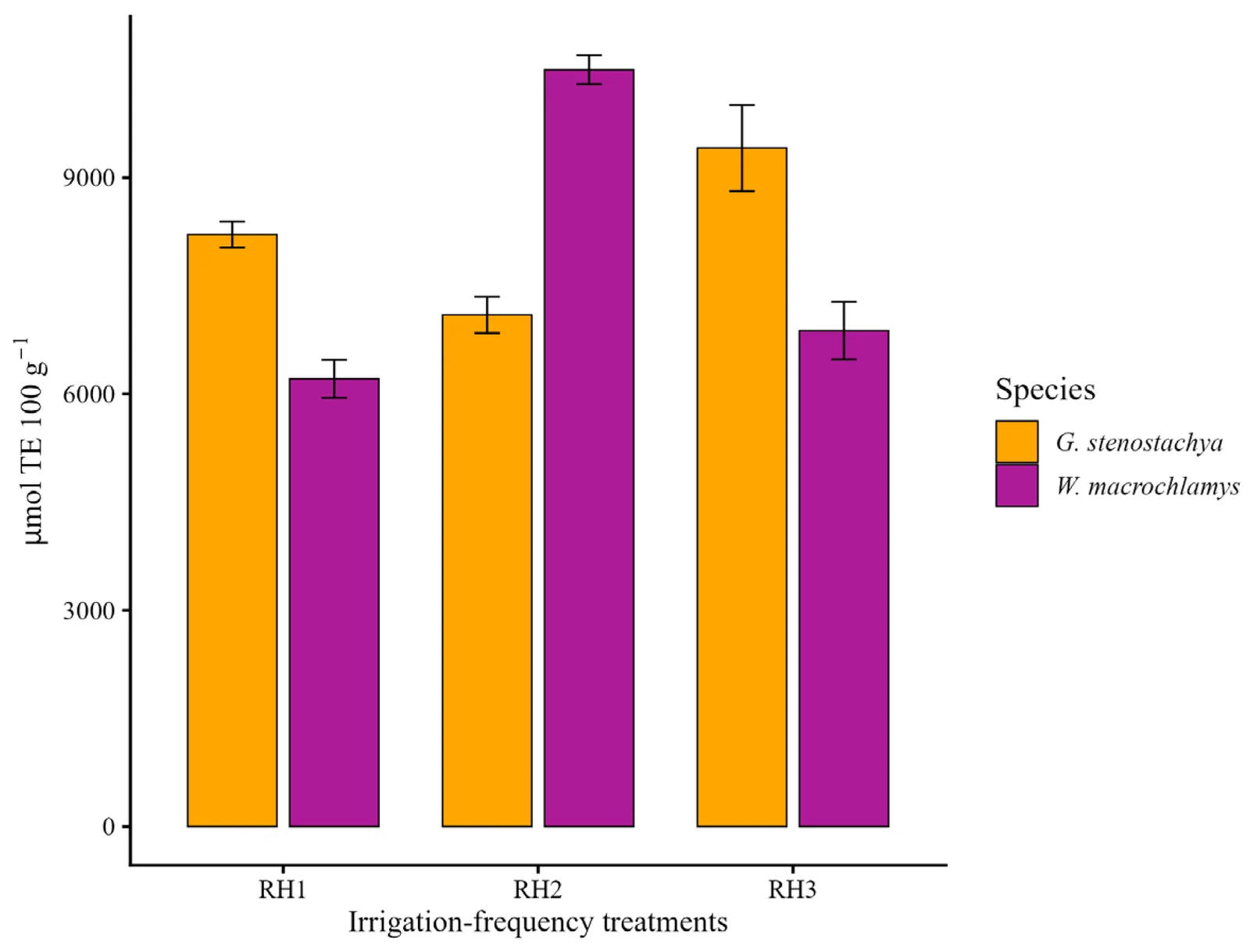
Effect of irrigation-frequency treatments on antioxidant capacity by ABTS assay in *G. stenostachya* and *W. macrochlamys*. Values are expressed as mean ± SD.

**Figure 5:**
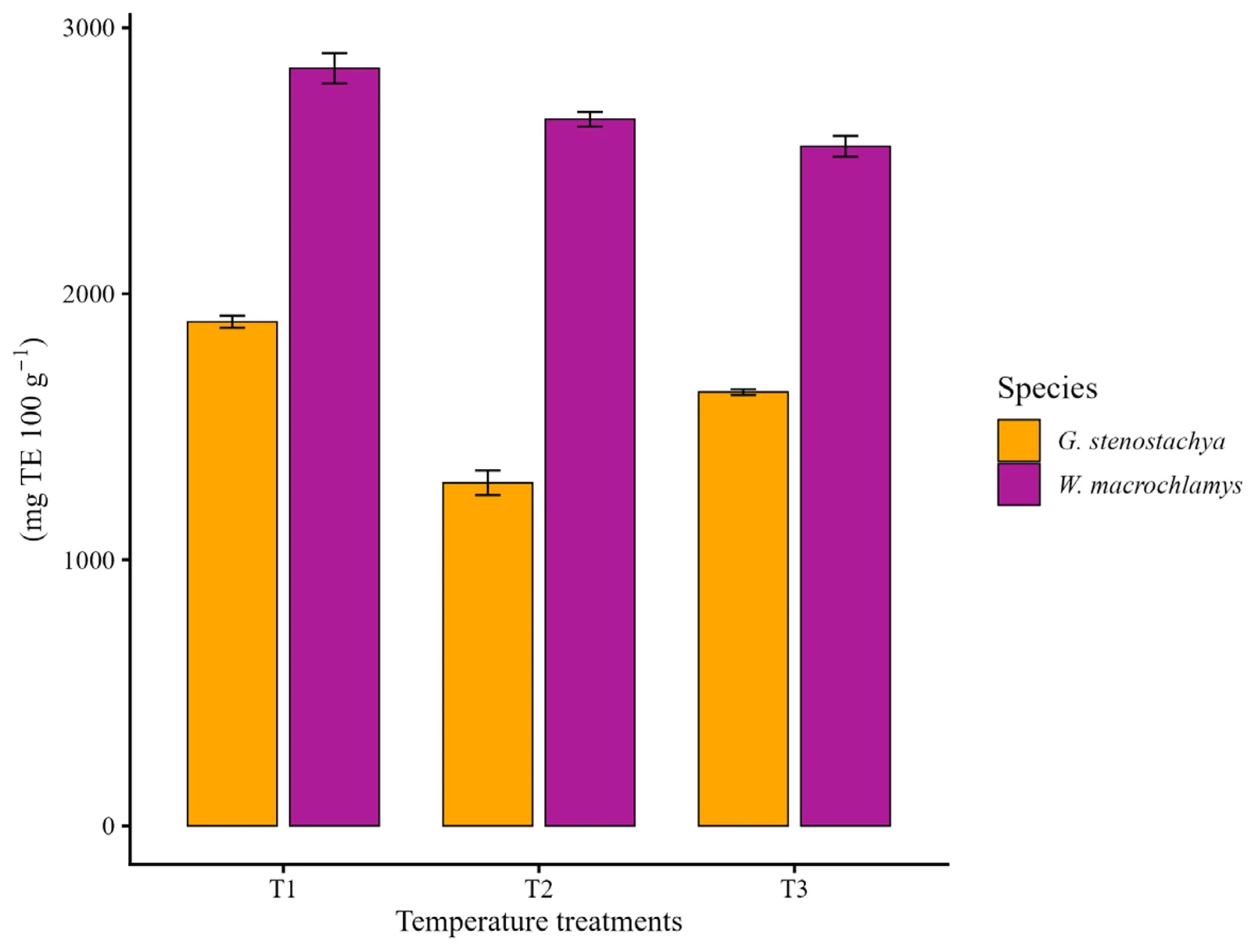
Effect of temperature treatments on antioxidant capacity by DPPH assay in *G. stenostachya* and *W. macrochlamys*. Values are expressed as mean ± SD.

**Figure 6:**
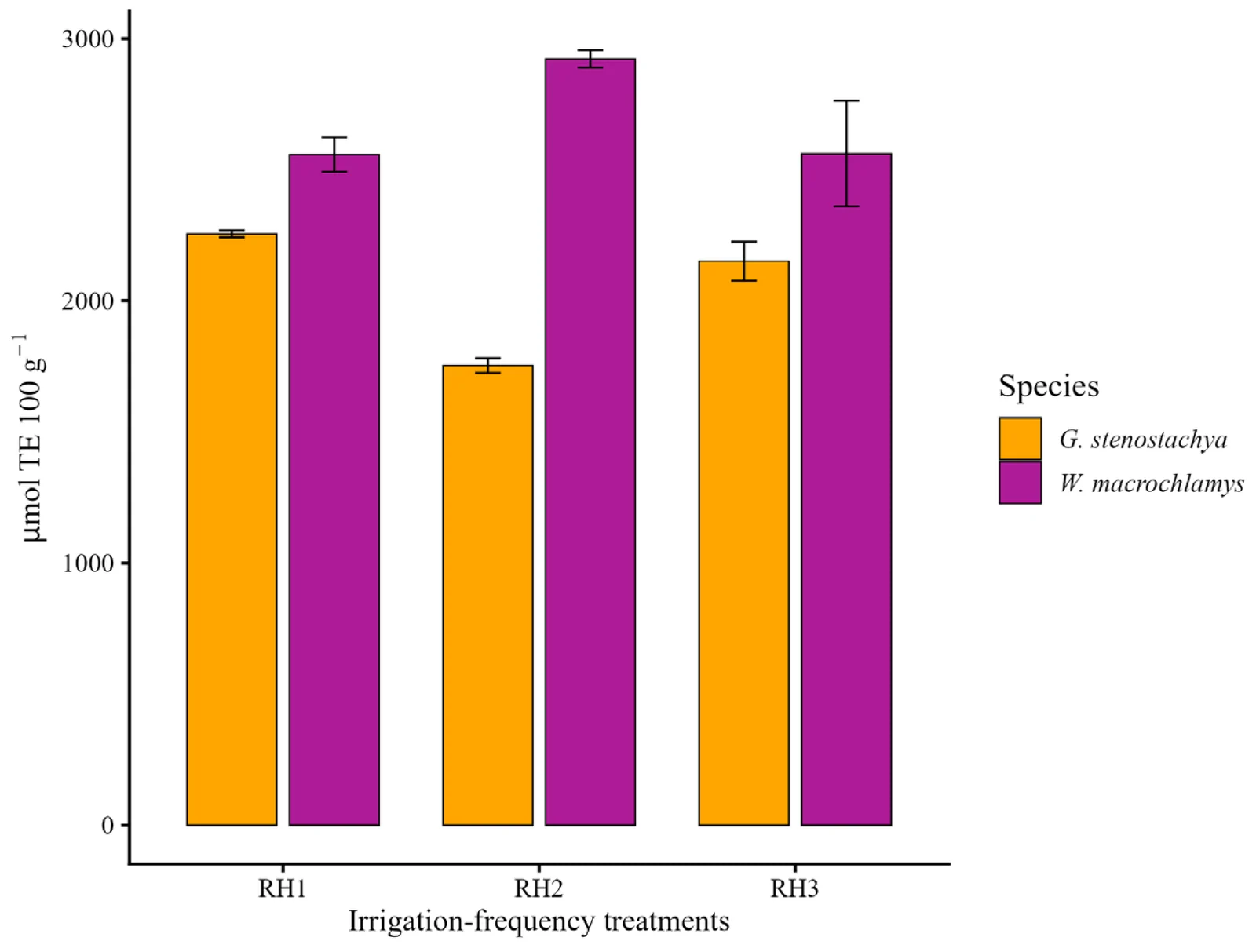
Effect of irrigation-frequency treatments on antioxidant capacity by DPPH assay in *G. stenostachya* and *W. macrochlamys*. Values are expressed as mean ± SD.

**Table 1:** Tentative compounds identified by GC-MS in hydroethanolic extracts of *G. stenostachya* and *W. macrochlamys* under temperature and irrigation-frequency treatments.

Sample/Condition	No.	Retention Time (min)	Compound	Match Factor (%)	Area (%)	Exp. RI	CAS No.	Reported Properties/Uses	Reference
Temperature treatments *G. stenostachya* T1	1a	3.699	Butanoic acid	91.086	1.42	823	107-92-6	Antiviral and antitumor activities	[15]
	2a	9.889	Limonene	90.022	0.29	1028	138-86-3	Antimicrobial, antibacterial, antifungal, and antiviral properties	[16]
	3a	10.358	Benzeneacetaldehyde	89.096	0.07	1043	122-78-1	Odorant; used as an olfactory cue and insect attractant	[17]
	4a	11.677	Fenchone	85.249	0.18	1082	1195-79-5	Antimicrobial and antibiofilm activities	[18]
	5a	13.455	(+)-2-Bornanone	99.765	93.07	1132	76-22-2	Insecticidal, antimicrobial, antiviral, anticoccidial, antinociceptive, anticancer, and antitussive activities	[19]
	6a	33.319	3-Methylene-7,11-dimethyl-1-dodecene	87.164	0.36	1847	1000432-21-6	Anticholinesterase activity	[20]
	7a	35.7533989	n-Hexadecanoic acid	92.282	0.76	1969	57-10-3	Antioxidant and antifungal activities	[21]
Temperature treatments *G. stenostachya* T2	1b	3.700	Butanoic acid	91.418	2.25	823			
	2b	8.373	Pentanoic acid	85.334	0.28	931	109-52-4	Modulates key processes such as satiety, insulin sensitivity, and inflammation	[22]
	3b	10.353	Benzeneacetaldehyde	85.562	0.19	1042			
	4b	13.457	(+)-2-Bornanone	99.702	90.60	1132			
	5b	26.285	2(4H)-Benzofuranone, 5,6,7,7a-tetrahydro-4,4,7a-trimethyl-	86.978	0.50	1519	15356-74-8	Potential multifunctional activity against Alzheimer’s disease	[23]
	6b	33.318	3-Methylene-7,11-dimethyl-1-dodecene	87.154	0.48	1847			
	7b	35.753	n-Hexadecanoic acid	89.301	1.70	1969			
Temperature treatments *G. stenostachya* T3	1c	3.700	Butanoic acid	93.150	0.74	823			
	2c	10.354	Benzeneacetaldehyde	92.435	0.17	1042			
	3c	11.675	Fenchone	85.040	0.07	1082			
	4c	12.166	α-Campholenal	86.770	0.61	1123	91819-58-8	Reported in *Juniperus phoenicea* essential oil with antimicrobial and anti-inflammatory activities	[24]
	5c	13.452	(+)-2-Bornanone	99.730	91.37	1132			
	6c	19.988	5-Ethenyl-2-methoxyphenol	85.816	0.14	1299	621-58-9	Associated with intense aromas during coffee storage	[25]
	7c	26.283	2(4H)-Benzofuranone, 5,6,7,7a-tetrahydro-4,4,7a-trimethyl-	85.499	0.19	1519			
	8c	33.322	Neophytadiene	87.262	0.22	1847	504-96-1	Anxiolytic activity and anticonvulsant effects	[26]
	9c	35.755	n-Hexadecanoic acid	90.403	0.61	1969			
Temperature treatments *W. macrochlamys* T1	1d	3.725	Butanoic acid	91.892	3.48	825			
	2d	10.352	Benzeneacetaldehyde	93.295	0.87	1042			
	3d	13.457	(+)-2-Bornanone	99.606	81.72	1132			
	4d	16.526	2,3-Dihydrobenzofuran	89.509	1.67	1213	496-16-2	Anti-HIV, anticancer, antibacterial, and antifungal activities	[27]
	5d	21.698	4-Hydroxybenzaldehyde	86.907	1.19	1353	123-08-0	Potential wound-healing agent	[28]
	6d	26.281	2(4H)-Benzofuranone, 5,6,7,7a-tetrahydro-4,4,7a-trimethyl-	90.473	0.96	1519	15356-74-8	Anticancer activity	[29]
	7d	35.754	n-Hexadecanoic acid	92.078	2.59	1969			
	8d	40.638	(9Z,12Z,15Z)-Octadeca-9,12,15-trien-1-ol	86.074	2.82	2095	506-44-5	Source of oligosaccharides	[30]
Temperature treatments *W. macrochlamys* T2	1e	3.700	Butanoic acid	87.913	2.41	823			
	2e	9.892	Limonene	87.165	0.36	1028	138-86-3	Antioxidant, anti-inflammatory, antitumor, antidiabetic, neuroprotective, and gastroprotective activities	[31]
	3e	10.358	Benzeneacetaldehyde	89.711	0.53	1043			
	4e	13.457	(+)-2-Bornanone	99.694	89.85	1132			
	5e	26.283	2(4H)-Benzofuranone, 5,6,7,7a-tetrahydro-4,4,7a-trimethyl-	88.107	0.53	519			
	6e	35.749	n-Hexadecanoic acid	86.433	1.02	1969			
Temperature treatments *W. macrochlamys* T3	1f	3.710	Butanoic acid	89.907	3.24	825			
	2f	9.889	Limonene	89.055	0.32	1028			
	3f	10.355	Benzeneacetaldehyde	88.232	0.55	1042			
	4f	13.456	(+)-2-Bornanone	99.672	89.98	1132			
	5f	16.643	2,3-Dihydrobenzofuran	85.616	1.20	1216			
	6f	35.751	n-Hexadecanoic acid	86.543	0.91	1969			
Irrigation-frequency treatments *G. stenostachya* RH1	1g	3.696	Butanoic acid	91.395	2.48	823			
	2g	9.887	Limonene	90.776	0.66	1028			
	3g	10.358	Benzeneacetaldehyde	93.011	0.35	1043			
	4g	13.456	(+)-2-Bornanone	99.633	89.19	1132			
	5g	19.972	5-Ethenyl-2-methoxyphenol	86.196	0.31	1299			
	6g	35.753	n-Hexadecanoic acid	90.043	2.15	1969			
Irrigation-frequency treatments *G. stenostachya* RH2	1h	10.356	Benzeneacetaldehyde	87.936	5.13	1043			
	2h	13.467	(+)-2-Bornanone	98.659	69.42	1132			
	3h	19.976	5-Ethenyl-2-methoxyphenol	85.928	4.00	1299			
	4h	35.753	n-Hexadecanoic acid	90.690	15.34	1969			
Irrigation-frequency treatments *G. stenostachya* RH3	1i	3.703	Butanoic acid	96.369	9.99	823			
	2i	10.347	Benzeneacetaldehyde	90.818	1.90	1042			
	3i	13.459	(+)-2-Bornanone	99.469	64.60	1132			
	4i	22.658	Vanillin	89.525	1.54	1384	121-33-5	Antioxidant, anti-inflammatory, neuroprotective, antifungal, antibacterial, and anticancer activities	[32]
	5i	26.281	2(4H)-Benzofuranone, 5,6,7,7a-tetrahydro-4,4,7a-trimethyl-	91.295	2.33	1519			
	6i	33.318	Neophytadiene	93.609	4.64	1847			
	7i	35.761	n-Hexadecanoic acid	96.057	11.35	1970			
Irrigation-frequency treatments *W. macrochlamys* RH1	1j	3.706	Butanoic acid	92.799	33.09	823			
	2j	10.355	Benzeneacetaldehyde	92.184	6.79	1042			
	3j	13.463	(+)-2-Bornanone	98.078	53.87	1132			
Irrigation-frequency treatments *W. macrochlamys* RH2	1k	3.702	Butanoic acid	88.316	9.23	823			
	2k	10.359	Benzeneacetaldehyde	89.588	1.89	1043			
	3k	13.460	(+)-2-Bornanone	99.364	81.38	1132			
	4k	33.317	Neophytadiene	86.284	1.93	1847			
Irrigation-frequency treatments *W. macrochlamys* RH3	1l	3.705	Butanoic acid	90.036	16.69	823			
	2l	10.356	Benzeneacetaldehyde	93.702	5.23	1043			
	3l	13.462	(+)-2-Bornanone	99.031	62.16	1132			
	4l	16.613	2,3-Dihydrobenzofuran	89.352	10.56	1215			
	5l	19.989	5-Ethenyl-2-methoxyphenol	85.976	1.48	1299			

## Data Availability

The original contributions presented in this study are included in the article. Further inquiries can be directed to the corresponding author.

## Supplementary Materials

The following supporting information can be downloaded at: https://www.mdpi.com/article/10.3390/molecules31193395/s1, Figure S1: Procedural blank chromatogram acquired under identical instrumental conditions; Figure S2. Representative total ion chromatogram (TIC) of the hydroethanolic leaf extract of *G. stenostachya* grown at 23 °C. Figure S3. Representative total ion chromatogram (TIC) of the hydroethanolic leaf extract of *G. stenostachya* grown at 26 °C; Figure S4. Representative total ion chromatogram (TIC) of the hydroethanolic leaf extract of *G. stenostachya* grown at 29 °C; Figure S5. Representative total ion chromatogram (TIC) of the hydroethanolic leaf extract of *W. macrochlamys* grown at 23 °C; Figure S6. Representative total ion chromatogram (TIC) of the hydroethanolic leaf extract of *W. macrochlamys* grown at 26 °C; Figure S7. Representative total ion chromatogram (TIC) of the hydroethanolic leaf extract of *W. macrochlamys* grown at 29 °C; Figure S8. Representative total ion chromatogram (TIC) of the hydroethanolic leaf extract of *G. stenostachya* under irrigation every 24 h (RH1) for 60 days; Figure S9. Representative total ion chromatogram (TIC) of the hydroethanolic leaf extract of *G. stenostachya* under irrigation every 48 h (RH2) for 60 days; Figure S10. Representative total ion chromatogram (TIC) of the hydroethanolic leaf extract of *G. stenostachya* under irrigation every 72 h (RH3) for 60 days; Figure S11. Representative total ion chromatogram (TIC) of the hydroethanolic leaf extract of *W. macrochlamys* under irrigation every 24 h (RH1) for 60 days; Figure S12. Representative total ion chromatogram (TIC) of the hydroethanolic leaf extract of *W. macrochlamys* under irrigation every 48 h (RH2) for 60 days; Figure S13. Representative total ion chromatogram (TIC) of the hydroethanolic leaf extract of *W. macrochlamys* under irrigation every 72 h (RH3) for 60 days.

## Abbreviations

The following abbreviations are used in this manuscript:

m a.s.l.	meters above sea level
TPC	Total phenolic content
GAE	Gallic acid equivalents
TE	Trolox equivalents
ABTS	2,2′-azino-bis(3-ethylbenzothiazoline-6-sulfonic acid)
DPPH	2,2-diphenyl-1-picrylhydrazyl
GC-MS	Gas chromatography–mass spectrometry
