# Supplementary material for: Temperature and Irrigation-Frequency Treatments Modulate Phytochemical Profiles and Antioxidant Capacity in Guzmania stenostachya and Werauhia macrochlamys
Source: Molecules. 2026 Sep 24;31(19):3395. doi: 10.3390/molecules31193395 (PMC13665858; doi:10.3390/molecules31193395)
Supplement: Supplementary file 1 [file molecules-31-03395-s001.zip › molecules-4545789-supplementary.pdf]

Supplementary Materials

Temperature and Irrigation-Frequency Treatments Modulate Phytochemical Profiles and Antioxidant Capacity in *Guzmania stenostachya* and *Werauhia macrochlamys*

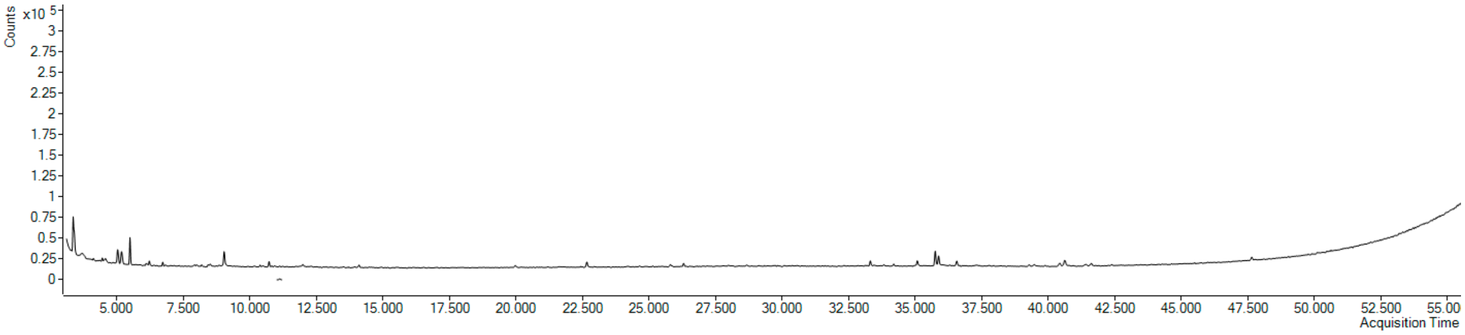

Figure S1. Procedural blank chromatogram acquired under identical instrumental conditions.

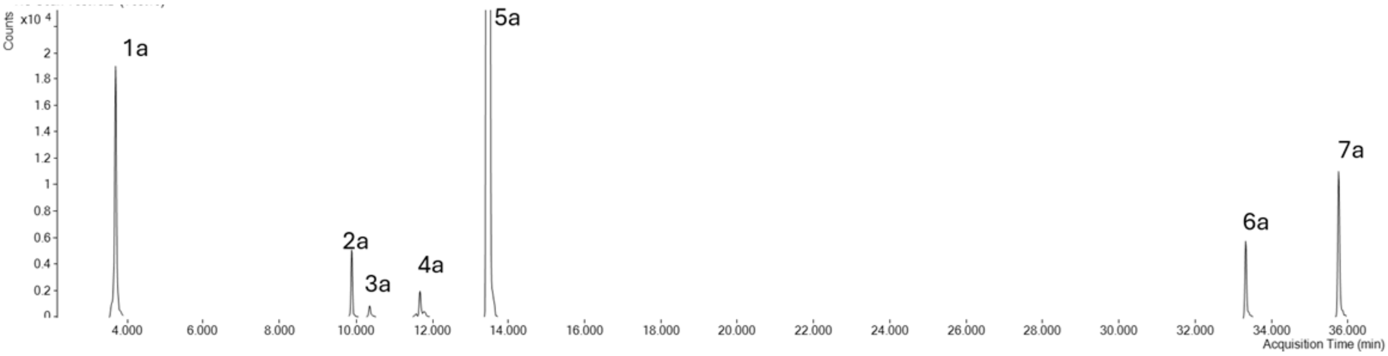

Figure S2. Representative total ion chromatogram (TIC) of the hydroethanolic leaf extract of *G. stenostachya* grown at 23 °C.

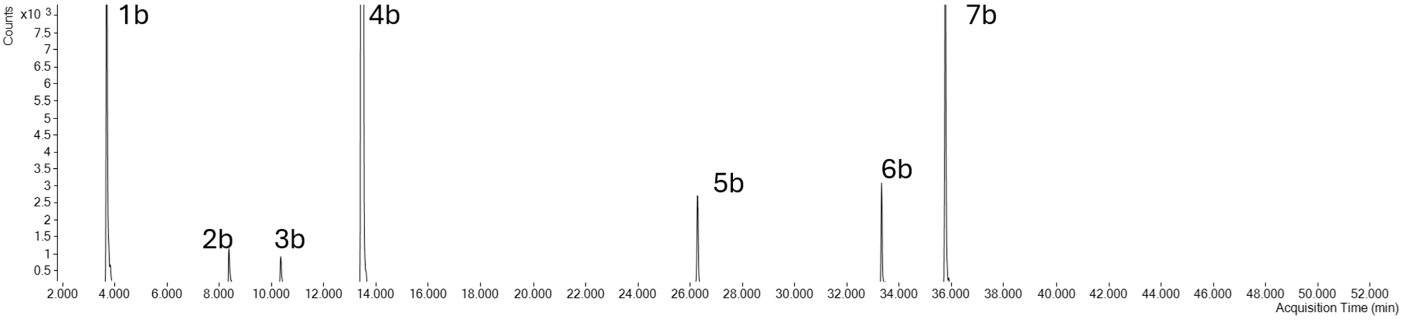

Figure S3. Representative total ion chromatogram (TIC) of the hydroethanolic leaf extract of *G. stenostachya* grown at 26 °C.

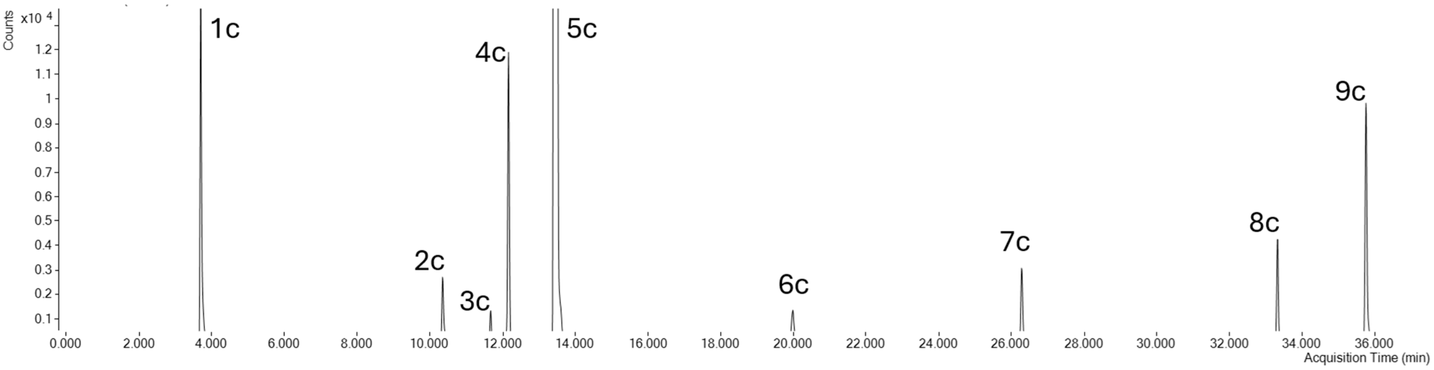

Figure S4. Representative total ion chromatogram (TIC) of the hydroethanolic leaf extract of *G. stenostachya* grown at 29 °C.

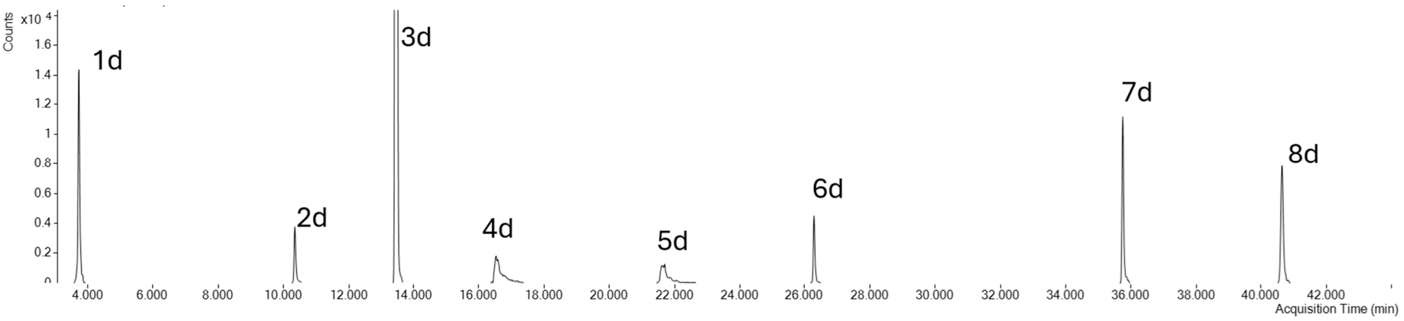

Figure S5. Representative total ion chromatogram (TIC) of the hydroethanolic leaf extract of *W. macrochlamys* grown at 23 °C.

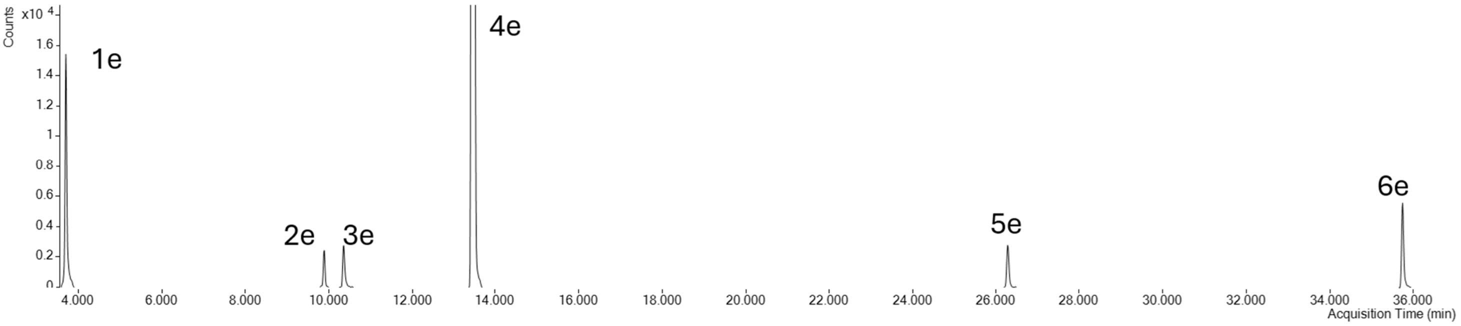

Figure S6. Representative total ion chromatogram (TIC) of the hydroethanolic leaf extract of *W. macrochlamys* grown at 26 °C.

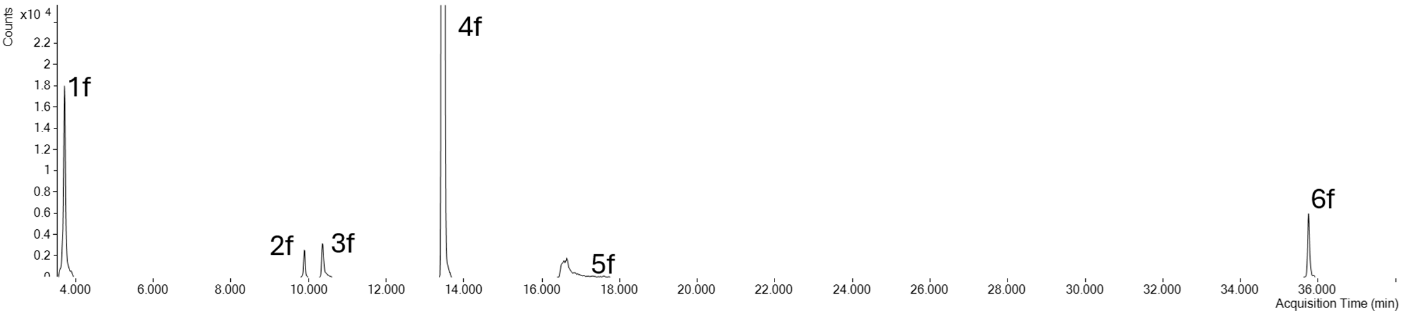

Figure S7. Representative total ion chromatogram (TIC) of the hydroethanolic leaf extract of *W. macrochlamys* grown at 29 °C.

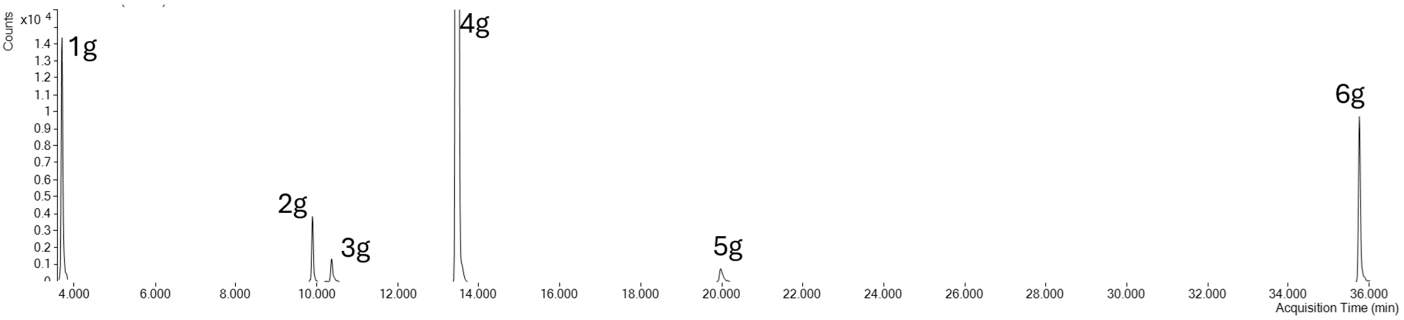

Figure S8. Representative total ion chromatogram (TIC) of the hydroethanolic leaf extract of *G. stenostachya* under irrigation every 24 h (RH1) for 60 days.

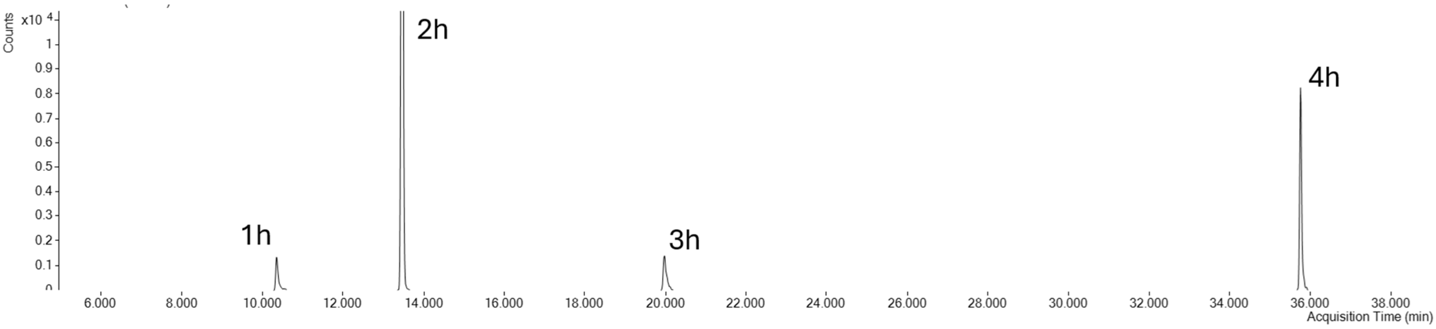

Figure S9. Representative total ion chromatogram (TIC) of the hydroethanolic leaf extract of *G. stenostachya* under irrigation every 48 h (RH2) for 60 days.

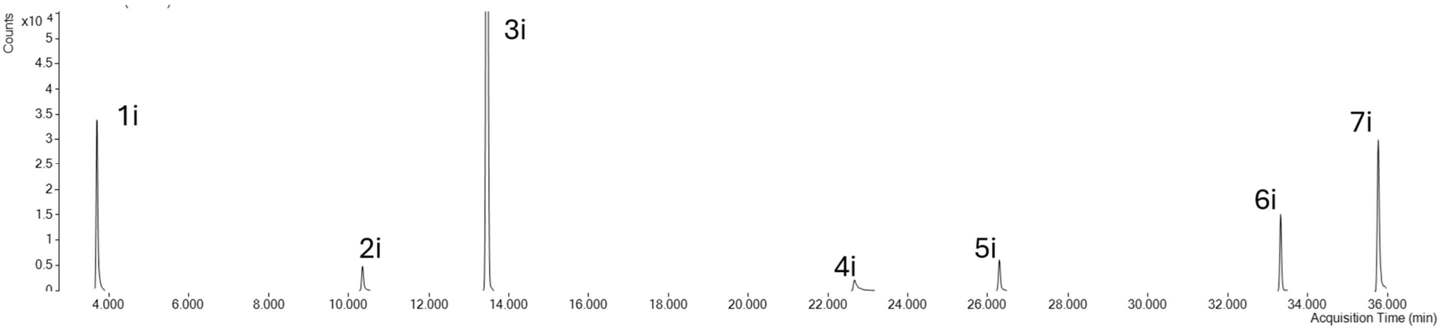

Figure S10. Representative total ion chromatogram (TIC) of the hydroethanolic leaf extract of *G. stenostachya* under irrigation every 72 h (RH3) for 60 days.

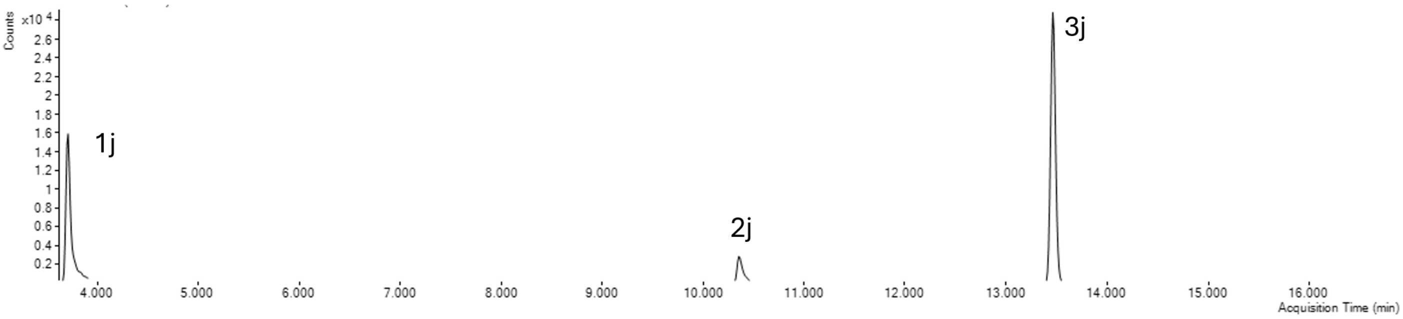

Figure S11. Representative total ion chromatogram (TIC) of the hydroethanolic leaf extract of *W. macrochlamys* under irrigation every 24 h (RH1) for 60 days.

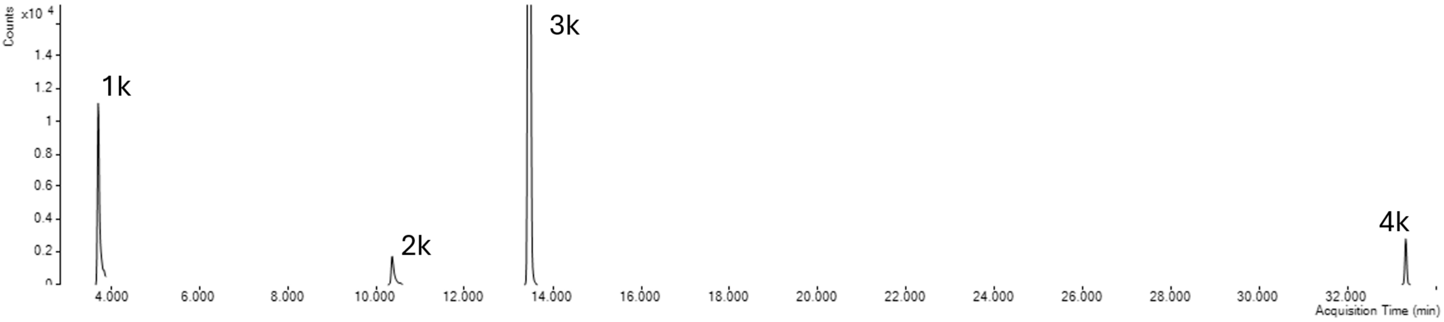

Figure S12. Representative total ion chromatogram (TIC) of the hydroethanolic leaf extract of *W. macrochlamys* under irrigation every 48 h (RH2) for 60 days.

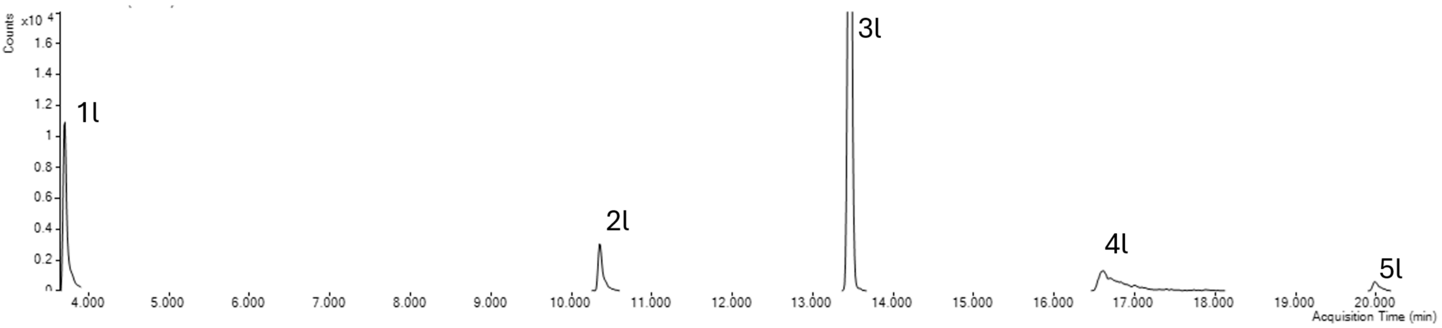

Figure S13. Representative total ion chromatogram (TIC) of the hydroethanolic leaf extract of *W. macrochlamys* under irrigation every 72 h (RH3) for 60 days.
